# Vertically integrated diffractive gratings on photonic crystal surface emitting lasers

**DOI:** 10.1038/s41598-021-82194-4

**Published:** 2021-01-28

**Authors:** Lih-Ren Chen, Kuo-Bin Hong, Hsiu-Ling Chen, Kuan-Chih Huang, Tien-Chang Lu

**Affiliations:** Department of Photonics, College of Electrical and Computer Engineering, National Chiao Tung University, Hsinchu City, 30010 Taiwan

**Keywords:** Engineering, Optics and photonics

## Abstract

We designed and fabricated a photonic crystal surface emitting laser (PCSEL) with vertically integrated diffractive optical elements on their top to study the mechanism of static beam steering on a single chip. The deflected output beam by the self-formed periodic ITO cladding layer of the PCSEL can be further steered by changing the grating period and azimuthal angle of the diffractive gratings relative to the photonic crystal. Through the analysis of photonic band structure and lasing characteristics, the periodic ITO structure is coupled to the photonic crystal band, whereas the integrated grating serves the diffractive function only. The findings pave the way for the design of PCSELs enabling single or multiple output beam with varying direction capability. This type of laser is regarded as an ideal light source for various applications, such as light detection and ranging and three-dimensional sensing systems.

## Introduction

Photonic-crystal surface-emitting lasers (PCSELs) are considered to be ideal light sources for applications such as light detection and ranging (Lidar) and three-dimensional (3D) sensing^1–5^. PCSELs exhibit a large output power on a single device, single wavelength, surface-emitting direction, circular beam shape, and small beam divergence. Moreover, due to the two dimensional (2D) distributed feedback lasing mechanism over the entire plane area^6–8^, the optical density of these lasers is relatively low to prevent a catastrophic optical damage at high output powers^9, 10^. The surface-emitting configuration also facilitates wafer level testing and (2D) integration of the laser array^11, 12^. In the current commercial Lidar or 3D sensing devices, laser light sources are combined with external optical and mechanical elements to steer the output beam for scanning^13, 14^. The resulting system is typically bulky and costly. Recently, beam-steering systems with chip-scale dimensions, such as optical phase arrays^15–19^, slow-light waveguide^20, 21^, tunable meta-surfaces^22–24^, and composite photonic-crystal (PC) structures^25–28^ have been proposed. However, the efficiency of these devices will be suffered due to its mechanism of light deflection. To reduce the output power loss at the interconnection of optical elements while maintaining the device size on the chip scale, we propose the use of vertically integrated diffractive elements on PCSELs for static beam steering. The designed structure is based on PCSELs with a low-cost indium-tin-oxide (ITO) cladding layer according to our previous report^2, 26^. The ITO layer exhibits a higher electrically conductive nature and a lower refractive index than typical semiconductors. This layer exhibits the function of current injection and optical confinement. A periodic structure is naturally formed in the ITO cladding layer as the thickness of the layer increases to 400 nm^26^. The original circular output beam in the surface normal direction is then split into two beams with deflection angles of 4–5° with respect to the vertical direction. The aforementioned mechanism is used to obtain vertically integrated optical diffraction elements on PCSELs for achieving beam steering and a high output power. In this study, various designs of vertically integrated diffractive gratings on PCSELs (VIDG-PCSELs) were investigated for determining the correspondence between the output beam deflection angle and the designed structure. The integrated structure has a negligible influence on the lasing threshold and spectra, and the beam direction is altered according to our expectations. The characteristics of the laser, such as the output-light power–current–voltage (L–I–V) curves, near and far field patterns, and PC band structures were also measured and analyzed.

## Results and discussion

**Figure 1:**
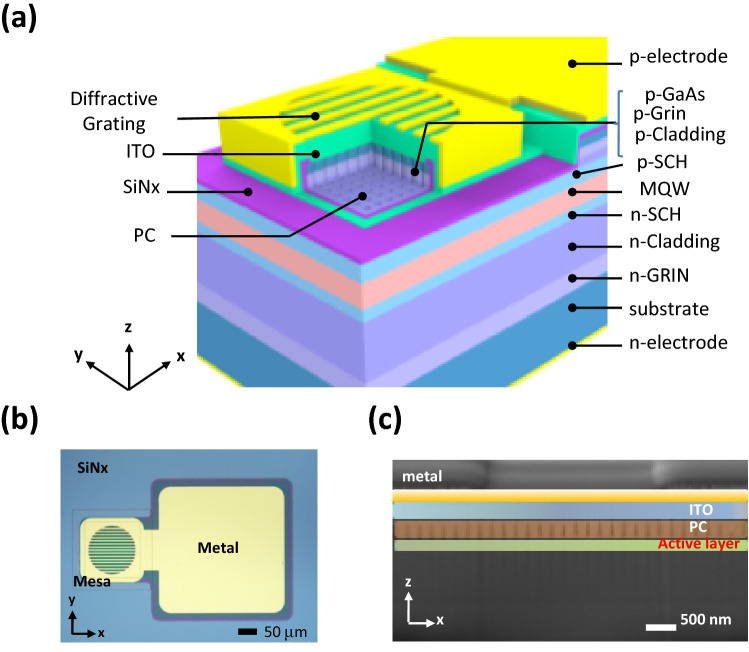
(**a**) Three-dimensional schematic diagram of a PCSEL with vertically integrated multiple diffractive elements. (**b**) The top-view image of the VIDG-PCSEL taken by an optical microscope. The metal grating is parallel to the x axis. (**c**) The cross-sectional scanning electron microscope image of the device with the metal grating and ITO periodic structure perpendicular to the x axis. The key layers are labeled and false-colored for easy identification. The ITO periodic structure is emphasized by blue and cyan colors according to the contrast of SEM image in the gray scale.

**Figure 2:**
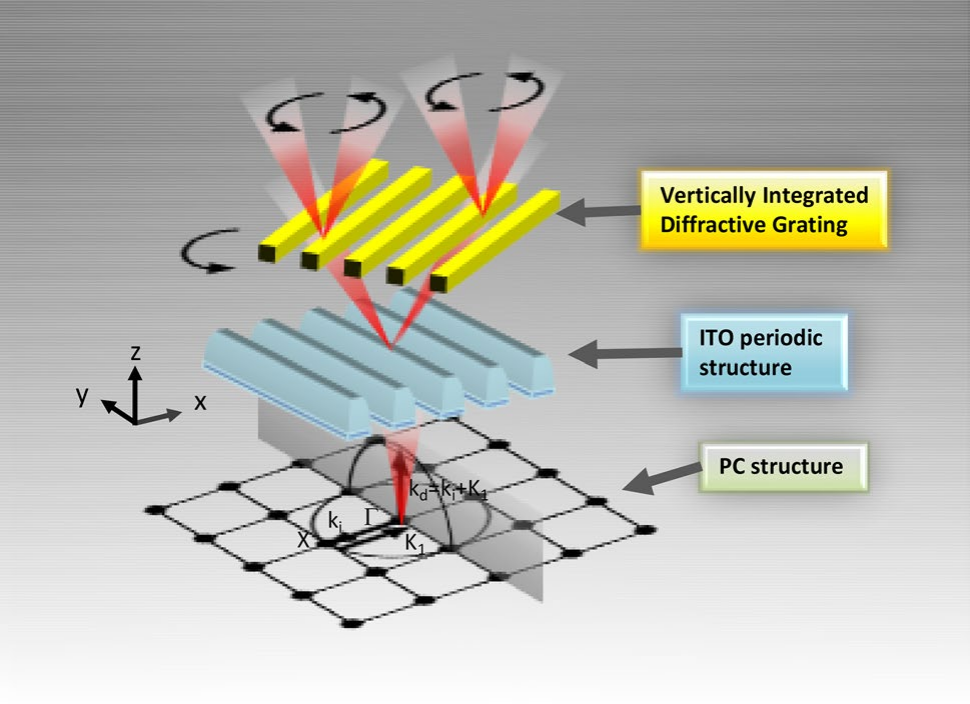
Illustration of the operation principle of VIDG-PCSEL is shown. The laser beam (in red color) emitted from the PC structure is split into two by the periodic structure of ITO layer and then deflected by the diffractive element made of metal grating.

Figure 1a displays the schematic of the VIDG-PCSEL structure. The major components of the structure are the epitaxial wafer, PC layer, ITO cladding layer, and vertically integrated diffraction structure. The square-latticed PC comprising of periodic circular air holes with a lattice constant (*a*) of 275 nm and a filling factor (FF) of 18% was designed for a lasing wavelength of 940 nm. A simple metal grating with a period of 8 μm and FF of 37.5% was applied as the optical diffraction element (Fig. 1b) to identify each optical mode without confusion. The azimuthal angles of the gratings, which are defined as the grating strip angles with respect to the *x*-axis (Fig. 1a), were varied from 0^o^ to 45° (0°, 30°, and 45°) to observe the corresponding changes in the output beam. The top-view image of the device captured using an optical microscope and the cross-sectional scanning electron microscopy (SEM) image of the device are displayed in Fig. 1b,c, respectively. Further details of the device structure are presented in the supplementary information. The operation principle of VIDG-PCSELs is illustrated in Fig. 2. The parameters of the PC were chosen to fit the second-order grating condition for forming an in-plane laser cavity and simultaneously allowing the in-plane laser light to be diffracted vertically by conserving the energy and momentum as displayed in Fig. 2. The laser beam emitted vertically from the PC structure was split into two by the periodic structure of ITO layer and then deflected by the diffraction element (metal grating). The far-field pattern was expected to change with the azimuthal angle.

**Figure 3:**
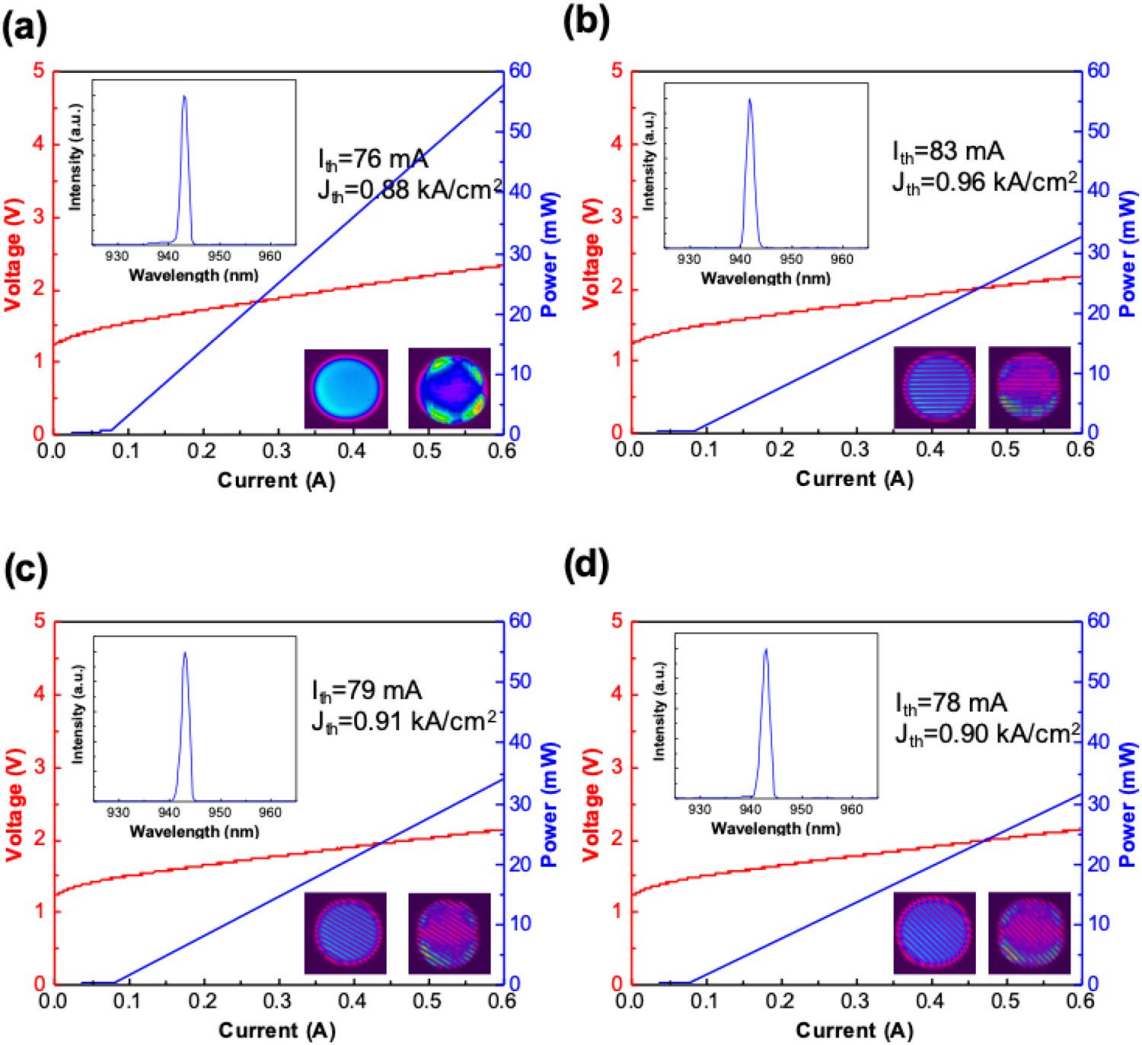
The L–I–V curve, threshold current, threshold current density, lasing wavelength and Near-field emission patterns (below and above threshold) of (**a**) PCSEL without grating structure, (**b**) VIDG-PCSEL with 0° grating, (**c**) VIDG-PCSEL with 30° grating and (**d**) VIDG-PCSEL with 45° grating, respectively.

Figure 3a displays the L–I–V curve of the control sample, which does not contain the metal grating structure. Figures 3b-d present the L–I–V curves of the samples with grating azimuthal angles of 0°, 30°, and 45°, respectively. In the following paragraph, the terms 0° grating, 30° grating, and 45° grating are used for the aforementioned samples, respectively. The threshold current (*I*_*th*_), turn-on voltage, and lasing wavelength of the four samples were nearly identical as illustrated in the Fig. 3. The threshold current density and turn on voltage ranged from 0.88 to 0.96 kA/cm^2^ and 1.24 to 1.26 V, respectively. The lasing wavelength is approximately 942 nm. This finding indicates that the integrated metal grating had a negligible influence on the lasing mechanism of the PCSEL and can be explained by the transverse optical field distribution presented in our previous report^26^. According to our previous report, the field penetration is negligible out of the ITO cladding layer. Therefore, the metal loss of the grating did not influence the laser threshold. The output power and the slope efficiency of PCSELs with metal grating structures were lower than those of the control sample due to the shadow effect of the metal grating. The insets at the lower right part of the Fig. 3 show the near-field emission patterns of corresponding devices measured at current of 0.8 $\times$ *I*_*th*_ (left figure) and 1.2 $\times$ *I*_*th*_ (right figure), respectively.

**Figure 4:**
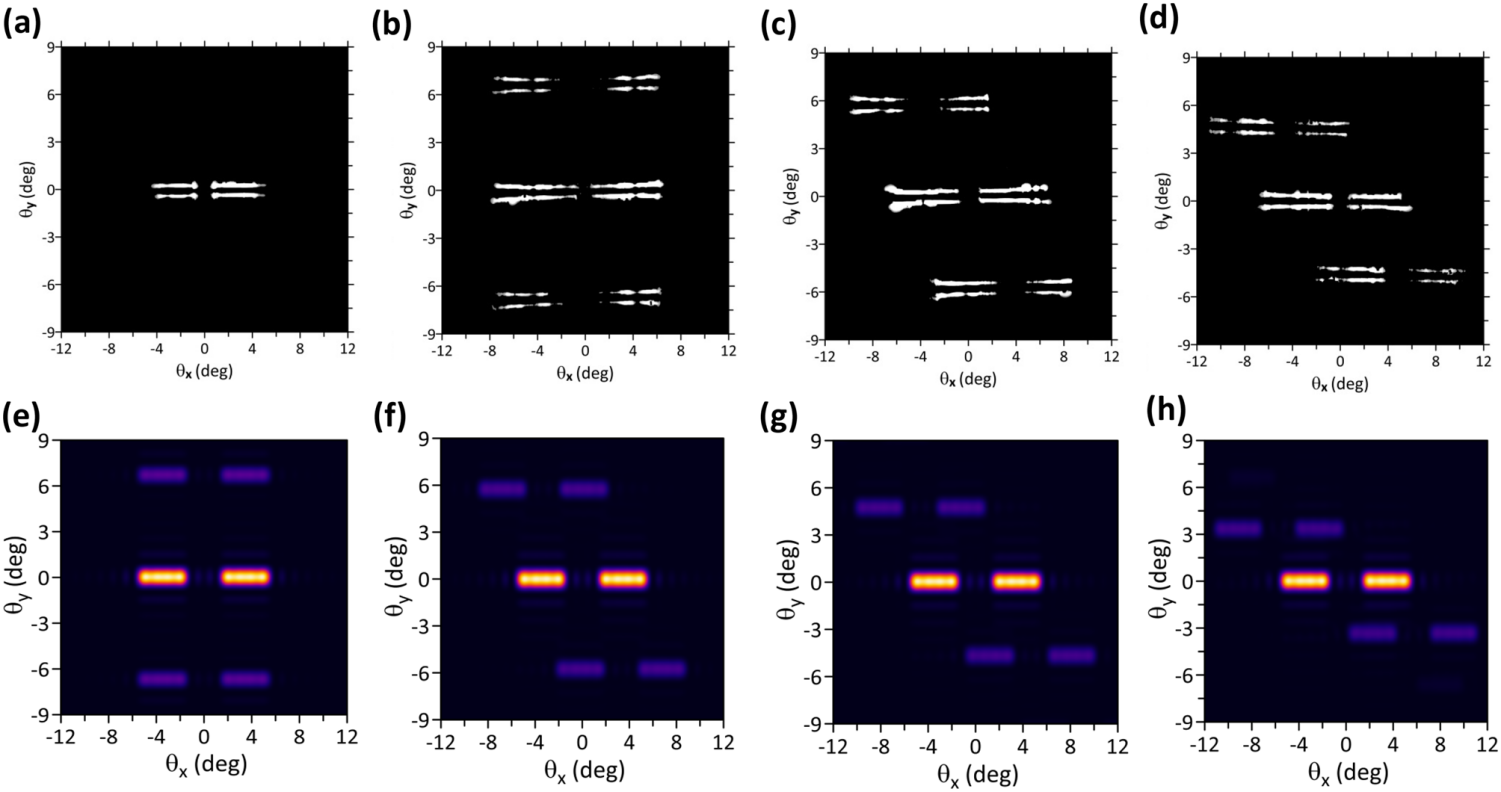
Far-field emission pattern of PCSELs operating above threshold (1.2 $\times$ I_th_). (**a**), (**b**), (**c**), and (**d**) display the measured far-field patterns of the VIDG-PCSELs of no grating, 0° grating, 30° grating, and 45° grating, respectively. (**e**), (**f**), (**g**), and (**h**) displays the simulated far-field patterns of 0° grating, 30° grating, 45° grating, and 60° grating, respectively.

The far-field patterns measured above the threshold (1.2 $\times$ *I*_*th*_) of the four samples are displayed in Fig. 4a-d. Four strip-like beams were observed from the far-field pattern of the PCSEL without the grating structure on top. The beam split in the *x* direction was caused by the naturally formed periodic structure in ITO as our previous report^26^. Because the naturally formed period of the ITO layer ranged from approximately 10 to 27 μm, the relatively broad divergence in the *x* direction resulted in strip-like patterns. The split in y direction of approximately 1° from Fig. 4a can be calculated by Fraunhofer diffraction equation from the near-field pattern shown in the inset of Fig. 3a which was most intense around the edge of the 100 μm circular aperture. The dimming emission at the center of near-field pattern was due to the destructive interference caused by the symmetric PC pattern as discussed in reference^29^. The grating structure used on the PCSELs resulted in two first-order diffraction beams in the ± y-direction (Fig. 4b-d). The separation of the zeroth and first-order diffraction beams was about 6.7 ^o^, which is in line with the calculation results obtained from the diffraction equation for the grating period of 8 μm. Moreover, the first-order diffraction beam shifted in the ± x-direction and the shifted diffraction angle was in accordance with the grating azimuthal angle. The simulation results of the far-field patterns corresponding to the device configurations with 0° grating, 30° grating, 45° grating, and 60° grating are displayed in Fig. 4e-h, respectively. Details of the simulation model are presented in the supplementary information. The simulation result revealed that the angle of the first-order diffraction beam shifted according to the grating rotation, which agreed with the experimental results. The experimental and simulation results proved the feasibility of the proposed design concept (Fig. 2), that is, the output beam can be deflected according to the rotational angle of the diffractive elements. The polarization and of the PCSELs are presented in supplementary. The electric field and near-field profile of PCSEL with periodic ITO structure are calculated and illustrated in supplementary.

**Figure 5:**
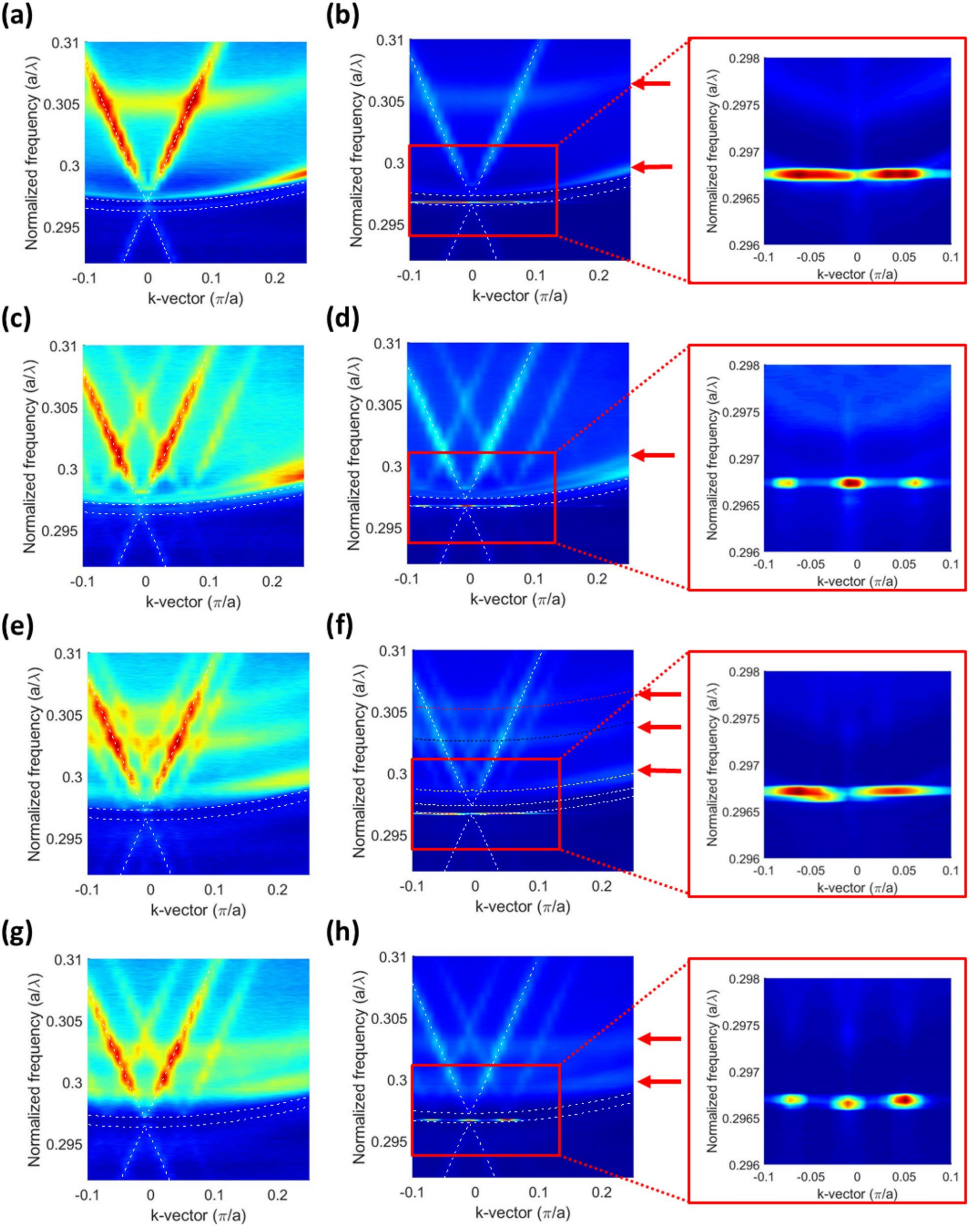
The photonic band structures measured by angle-resolved electroluminescence. (**a**) and (**b**) represents the PC band structures measured along the *x* direction (directions defined in Fig. 1) of the PCSEL without grating structure driving below (0.8 $\times$ I_th_) and above (1.2 $\times$ I_th_) threshold conditions, respectively. (**c**) and (**d**) display the PC band structures of the VIDG-PCSEL with 0° grating measured along the *y* direction. (**e**) and (**f**) show the PC band structures measured along the *x* direction of 30° grating device below and above threshold, respectively and (**g**) and (**h**) show the photonic band structures of the same device of (**e**) measured along the *y* direction. The insets show the magnified lasing modes around the Γ point of each band structures. The dashed lines represent the PC band structures of the square lattice and guided modes as detailed in the supplementary information.

For further analysis of the influence of periodic ITO structure and the diffractive elements on the photonic band structure of the devices, Angle-resolved electroluminescence (AREL) analysis was conducted (Fig. 5). The AREL measurement was taken at driving current below (0.8 $\times$ *I*_*th*_) and above (1.2 $\times$ *I*_*th*_) threshold conditions at room temperature. Figure 5a,b represent the PC band structures measured along the *x* direction (direction defined according to Fig. 1 or along the Γ−X direction in the reciprocal space of the square lattice) of the PCSEL without grating structure on top to investigate the influence of the periodic ITO structure at an operating current below and above the threshold value, respectively. The inset of Fig. 5b represents a magnified lasing peak around the Г point, and the two lasing peaks separate due to the diffractive effect of the ITO periodic structure. Figure 5c,d displays the PC band structures of the 0° grating VIDG-PCSEL device measured along the *y*-direction (the same Γ–X direction in the reciprocal space of square lattice). The aforementioned parts of Fig. 5 clearly present the band shift due to the metal grating. The PC-band along the *x*-direction not shown here exhibits the same result as that without grating, in which the effect of ITO periodic structure rather than the metal grating dominates. The shift caused by the grating momentum around 0.06 (π/a), which was measured using the graph presented in Fig. 5d, agrees with the calculation from the metal grating period (Λ = 8 μm).

Figures 5e-h represent the PC band structure of the 30° grating VIDG-PCSEL device under the same measurement condition as that for 0° grating counterpart. Figure 5e,f represents the band structures measured along the *x*–direction below and above threshold, respectively. Figure 5e reveals the effect of grating momentum on the band structure. The shift in the photonic band in the range of 0.03–0.05 (π/a), which was measured from the graphs presented in Fig. 5e,f, agreed well with the shift calculated from the rotated metal grating period (Λ_*x*_ = 8 μm/sin (30°) = 16 μm) when taking shift of 0.035 (π/a) into calculation. Because this period is similar to that of the ITO periodic structure, the lasing signals presented in the inset of Fig. 5f are similar to those in Fig. 5b. The shift in the band diagram for the *y-*component of grating momentum, which was measured from Fig. 5g was 0.062 (π/a). This shift agreed well with the shift calculated from the rotated metal grating period (Λ_*x*_ = 8 μm / cos (30°) = 9.23 μm). Some bands with relatively small slopes at high frequencies indicated by red arrows in the Fig. 5. are the higher-order modes induced by large effective indices. The analysis of the higher-order modes is presented in the supplementary information. The band structures without anti-crossing indicate that the grating structure was not strongly coupled with the PC band; hence, the design of the diffractive element can be free from the consideration of the lasing characteristics.

From the band diagram shown in Fig. 5b, we can clearly see the disappearance of the zeroth-order diffraction pattern^26^, which indicates the ITO periodic structure coupled to the PC band structure. In contrast, the band diagram of the PCSEL with additional diffractive grating shown in Fig. 5d, h), the zeroth-order diffraction pattern is clearly observed. Also, from the measurement results for *I*_*th*_ and the lasing wavelength, we ensure the additional diffractive grating was not coupled to the PC. The additional grating decoupled from PCSEL is favorable because the lasing characteristics such as *I*_*th*_, output power, and lasing wavelength is not influenced by the various design of additional grating. Moreover, the additional diffractive grating can further deflect the beam to a larger angle for which a nanometer scale surface structure is required. In this study, we applied metal grating as diffractive elements to obtain the intuitive information of the photonic band structure, however, the zeroth-order diffraction is undesirable for practical application. To annihilate the zeroth-order diffraction, we propose the sawtooth-shape amorphous silicon (α-Si) grating which can be fabricated by typical photolithography and thin film processes. The simulation reveals that the intensity of first-order diffraction is 57.1 and 9.5 times stronger than the zeroth-order and second-order. The grating structure and numerical simulation are described in the supplementary information. Another practically unfavorable strip-like lasing pattern that elongated in *x* direction was caused by the variation in the naturally formed grating periods of the ITO layer. A 200-nm ITO layer prepared through the sputtering process should be considered to obtain a flat surface and optimal conductivity and a subsequent photolithographic process can be used to define the periodic structure.

## Conclusions

The structural design and fabrication method of VIDG-PCSELs were demonstrated in this study, and the beam deflection mechanism was discussed. By combining grating period and grating azimuthal angle, PCSELs array of 2D steering of the laser beam capability can be achieved. The distinct diffraction function of the integrated gratings on the PCSEL increased the degree of freedom of the design. The laser beam shape can be further improved by eliminating the periodicity fluctuation of the ITO layer. The efficiency of the laser can be improved by applying asymmetric PC structures to eliminate the destructive interference as well. The metal grating which resulted in huge absorption loss of laser beam can be replaced by transparent material such as amorphous silicon proposed in supplementary. The designed laser with high power, single mode operation, and beam steering capability is very promising for various applications.

## Methods

The designed epitaxial structure was grown by metal–organic chemical vapor deposition (MOCVD) method on the GaAs substrate. The PC lattice was fabricated by e-beam lithography and Inductively Coupled Plasma (ICP) etching process. The ITO layer was deposited by the e-gun evaporator at substrate temperature of 300 °C. The diffraction element with the electrodes was deposited by the e-gun evaporator at room temperature.

The L–I–V curves were measured under the following driving conditions: pulse width, 1 μs; duty cycle, 0.1%; and temperature, 300 K.

The simulations were performed using COMSOL Multiphysics, a commercial Finite Element Method. (FEM) based simulation software.

For the energy-wave number (*E*-*k*) diagram measurement, emission spectra at different emission angles were collected by the collimator which moved along the Γ–X direction in the reciprocal space of the square lattice. The measurement angle is ranging from − 10° to 30°, corresponding to the normalized in-plane *k* vector range of − 0.1 (π/a) to 0.3 (π/a), with sampling step of 1°.

## Supplementary Information

Supplementary Information.
