# Supplementary material for: Vertically integrated diffractive gratings on photonic crystal surface emitting lasers
Source: Sci Rep. 2021 Jan 28;11:2427. doi: 10.1038/s41598-021-82194-4 (PMC7844237; doi:10.1038/s41598-021-82194-4)
Supplement: Supplementary file 1 — Supplementary Information. [file 41598_2021_82194_MOESM1_ESM.docx]

### Supplementary information

**Vertically integrated diffractive gratings on photonic crystal surface emitting lasers**

Lih-Ren Chen, Kuo-Bin Hong, Hsiu-Ling Chen, Kuan-Chih Huang, and Tien-Chang Lu*

Department of Photonics, College of Electrical and Computer Engineering, National Chiao Tung University, Hsinchu City 30010, Taiwan. +886-3-5131234

* timtclu@mail.nctu.edu.tw

**Detailed parameters of PCSELs**

The epitaxy structure followed our previous design^s1,1s2^, which was optimized to obtain optimal optical confinement factors for multiple quantum wells (MQWs) and PC layers. The designed epitaxial structure along the growth direction consisted of a 100-nm thick graded index (GRIN) n-Al_x_Ga_1−x_As (x =0~0.4) layer, a 980-nm thick n-Al_0.4_Ga_0.6_As cladding layer, a n side GaAs separate confinement heterostructure (SCH) layer, a three-pair InGaAs/GaAs MQWs active region targeting the emission wavelength of 940 nm , a p side GaAs SCH layer, a 100-nm thick p-Al_0.4_Ga_0.6_As cladding layer, a 100-nm thick GRIN pAl_x_Ga_1−x_As (x =0−0.4) layer, and a 100-nm thick p+ GaAs contact layer as depicted in Fig.1(a) in main text.

The PCSEL device was fabricated on the epitaxial wafer by the following processes: PC-layer formation, mesa etching, passivation deposition (to create a current injection aperture), ITO deposition, and diffractive structures together with conducting metal. The square-latticed PC comprising of periodic circular air holes with a lattice constant (*a*) of 275 nm and a filling factor (FF) of 18% was designed for a lasing wavelength of 940 nm. The PC layer with a total area of 125 × 125 μm^2^ was first defined using an e-beam lithography system and then etched with a p+ GaAs contact layer, p-GRIN layer, and p-AlGaAs cladding layer for 250~260 nm by using an inductively coupled plasma reactive ion etching system. Next, a 145 × 145 μm^2^ square mesa was fabricated through the wet-etching method to constrain the current flow. To prevent current leakage and further restrict current injecting into the desired PC region, a silicon nitride (SiN_x_) layer deposited through plasma-enhanced chemical vapor deposition was applied to serve as a passivation and current confinement layer. Moreover, a circular aperture with a diameter of 100 μm was left open for carrier injection. A 400-nm-thick ITO layer that served as the cladding layer and current conduction layer simultaneously was then deposited through an electron gun (e-gun) evaporator. Subsequently, the diffraction element as described in main text with the electrodes was also deposited by the e-gun evaporator to complete the entire fabrication process.

The lattice constant and air hole diameter of photonic crystal is detailed in scanning electron microscopy (SEM) picture of Fig. S1(a). The measured lattice constant and air hole diameter are 280 nm and 90 nm, respectively, which is consistent with our designed value. The cross-section view of photonic crystal (PC) structure shown in Fig. S1(b) depicted the steepness of the air hole and etching depth of around 255 nm that both properly meet our requirement. The SEM picture was taken from the sample of the wafer for PCSEL device before the ITO deposition.


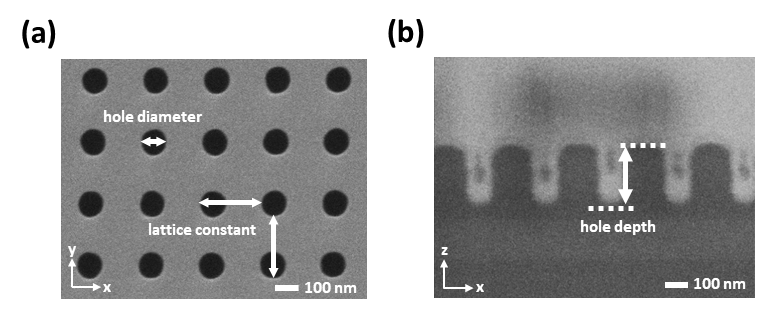


**Figure S1 |** (a) The top-view image of PC structure by SEM. The lattice constant and the air hole diameter are characterized before the ITO cladding layer deposition. (b) The cross-section view of the PC structure by SEM.

**Polarization of the PCSELs**

The polarization direction is basically dominated by the PC combining ITO structures and is not affected by the top metal grating, which is another proof that the diffraction function of the integrated gratings on the PCSEL increased the degree of freedom of the design.

**Figure S2 |** The polarization of the PCSELs without top gratings and with gratings along different directions.

**Simulation model for far field**

The simulation of the far-field patterns of vertically integrated multiple diffraction photonic crystal lasers shown in Fig. 5. (e)-(h). The light source incident to the grating structure of 8-μm-period is set to emulate the strip-like output beam of the PCSEL reported previously.^s1^ The strip-like pattern is simulated by the superposition of linearly polarized plane waves with incident angles of ±2°, ±3°, ±4°, and ±5°, respectively. The simulation is based on three-dimensional model, and the rotation of the grating structure is set according to the real case. The far-field pattern was obtained by calculation according to Fraunhofer diffraction theory.

**Simulation model for PC band structure**

In order to identify the higher order modes, we first consider the effective index of transversely guided modes of higher orders, then follows by calculating the band structure by using these effective indexes.^1-3^ The obtained effective index is 3.354 and the filling factor of PC airhole is 18%, the parameter are applied to calculate the effective index of the air hole region according to above mentioned references. The period of PC is 275 nm as described in the main text. Once the band structure is obtained, the measured band structure is fitted by the calculated one to determine the mode of oscillation. The result is shown in Fig. S**3**, in which the grating 30^o^ VIDG-PCSEL is illustrated. The higher order modes shown as red, black and yellow dashed lines in Fig. S**3**, according to our identification, are the transverse higher order counterparts of the guided modes.


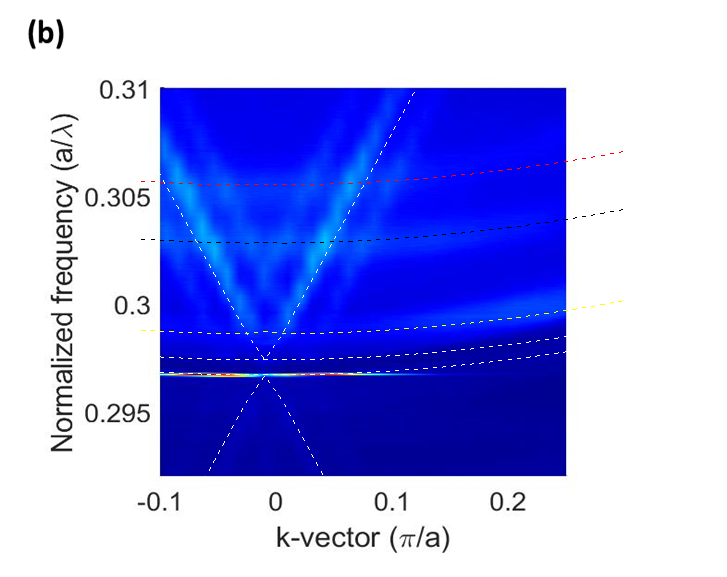


**Figure S3 |** The identification of higher order modes for VIDG-PCSEL operating above threshold.

**Illustration of simulated electric field**

Figure S4 depicted the structure of the PCSEL device with periodic ITO structures for the simulation of electric field distribution and the lasing mode profile.

**Figure S4** | (a) 3D schematic drawing of a PCSEL including a 40 nm depth ITO notch. Lateral sizes of the PCSEL 3D model contains 30×4 periods of photonic crystal. The color map in Fig. S4(b-d) shows the normalized electric field $\left| E \right|$ of TE polarized lasing mode. (b) The x-y cross-section plot of lasing mode $\left| E \right|$ cut at distance of quarter wavelength above the ITO grating. (c) The x-y cross-section plot of lasing mode $\left| E \right|$ cut at the middle of MQW layer. (d) The x-z cross-section plot of lasing mode $\left| E \right|$. Although Figs. S4(b)-(d) are expressed in different color scales, the lasing mode is well confined at MQW as shown in Fig. S4(d). Fig. S4(c) obviously shows the fundamental Г band edge mode B which accompanies a high quality factor of 2469.2 and the resonant wavelength of 942 nm that is in good agreement with experiment. The perpendicular white lines in Fig. S4(b) and (c) indicate the boundary of ITO notch which demonstrates this near field of PCSEL is adjusted by the ITO grating that will further yield small-angle light emission.

**Structure and simulation** **of the sawtooth-shape dielectric grating**

The proposed asymmetric grating structure is shown in Fig. S5(a), for which the periodic condition was applied along the *x* direction for the simulation. The amorphous silicon (α-Si) grating structure was deposited on the ITO cladding layer with grating period of 3490 nm (Λ = 3490 nm), and the shape projected on the *x*-*z* plane formed a right triangle with height of 453.8 nm.

The circular polarized plane wave was incident from the ITO side to analyze the phase change and the far field pattern of the transmitted electric field. Fig. S5(b) displays the propagation wave front of the electromagnetic field traveling through the grating structure, from which the deflection of the propagation direction is observed. Fig. S5(c) depicted the far field emission pattern calculated by Fraunhofer diffraction integral. The dominate peak appeared at 15.6^o^ and the intensity of first-order diffraction is 57.1and 9.5 times stronger than the zeroth-order and second-order from the simulation. The structure is feasible from simulation and fabrication from our analysis and the sample preparation is ongoing.

**
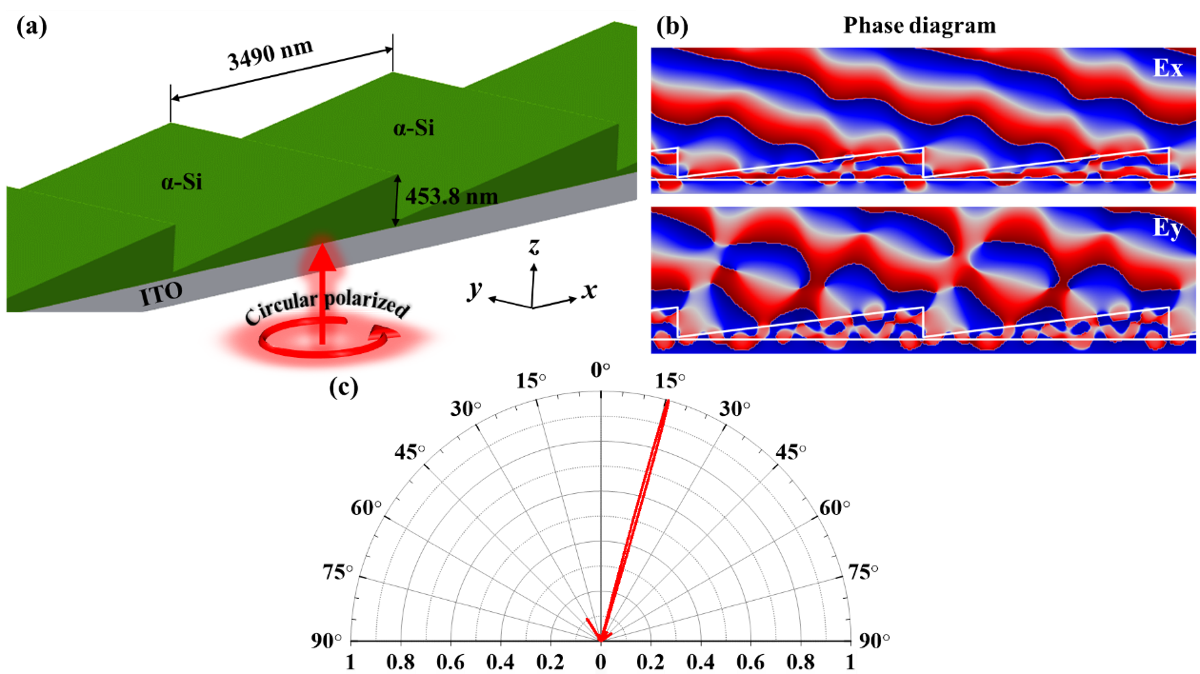
**

**Figure S5 |** (a) Illustration of the sawtooth-shape amorphous silicon (α-Si) grating. (b)The phase diagram of electric filed. (c) The polar plot of far field emission pattern.

**Reference**

1 Chiu, H.-L., Hong, K.-B., Huang, K.-C. & Lu, T.-C. Photonic Crystal Surface Emitting Lasers with Naturally Formed Periodic ITO Structures. *ACS Photonics* **6**, 684-690, doi:10.1021/acsphotonics.8b01530 (2019).

2 Huang, S. C. *et al.* Design of photonic crystal surface emitting lasers with indium-tin-oxide top claddings. *Applied Physics Letters* **112**, doi:Artn 06110510.1063/1.5016442 (2018).

3 Imada, M., Chutinan, A., Noda, S. & Mochizuki, M. Multidirectionally distributed feedback photonic crystal lasers. *Physical Review B* **65**, doi:10.1103/PhysRevB.65.195306 (2002).
